# Syphilis among people with HIV infection in southern Ethiopia: sero-prevalence and risk factors

**DOI:** 10.1186/s12879-015-0919-7

**Published:** 2015-04-17

**Authors:** Techalew Shimelis, Kinfe Lemma, Henock Ambachew, Endale Tadesse

**Affiliations:** Department of Medical Laboratory Science, Hawassa University, P. O. Box 1560, Hawassa, Ethiopia; Department of Internal Medicine, Hawassa University, P. O. Box 1560, Hawassa, Ethiopia

**Keywords:** Prevalence, Syphilis, HIV, Risk factor

## Abstract

**Background:**

Syphilis facilitates both HIV (human immunodeficiency virus) transmission and acquisition, reflecting the complex interplay between the two infections. Scarce information exists regarding syphilis epidemiology in Ethiopian context. Thus, this study determined the sero-prevalence of syphilis and associated risk factors in people with HIV infection.

**Methods:**

A cross-sectional study was conducted at Hawassa Referral Hospital, southern Ethiopia from January to May, 2014. A consecutive 993 HIV–infected participants were studied; but individuals under 15 years of age or treated for syphilis or those with a CD4+ T–cell count below 50 cells/μl were excluded. Structured questionnaires were used to collect data on socio-demography and potential risk factors for syphilis. Moreover, blood samples were collected from all participants and screened for syphilis using rapid plasma reagin (RPR) test, and those found sero-positive were confirmed using treponema pallidum haemagglutination assay (TPHA).

**Results:**

The sero-prevalence of syphilis was found to be 7.3% (95% CI 5.7– 9%). The rate of infection was significantly higher among participants who were ART (antiretroviral therapy) naive (odds ratio (OR) = 2.2; 95% CI 1.22 – 4.1), men (OR = 2.2; 95% CI 1.22 – 3.87), older than 50 years of age (OR = 3.9; 95% CI 1.45 – 6.94), had only primary school level education (OR = 7.8; 95% CI 2.63 – 23.2) and had a history of blood transfusion (OR = 3.9; 95% CI 1.5 – 10.4).

**Conclusion:**

The high prevalence of syphilis among HIV-infected population warrants integrating syphilis screening with HIV care to limit the clinical consequences of untreated syphilis as well as its adverse impact on HIV transmission.

## Background

Syphilis, one of the oldest diseases caused by the bacterium *Treponema pallidum*, has been a major public health problem worldwide. An estimated 12 million people globally have been infected; of which, almost two-thirds are in sub-Saharan Africa and south/southeast Asia [1]. Transmission of syphilis is mainly through unprotected sex and vertical transmission from infected mother to the child [1,2]. The risk of transmission through blood transfusion is negligible in contexts that ensure improved donor selection and testing as well as refrigerated blood components are transfused [3]. The possibility of parenteral transmission of syphilis among individuals with drug-using behaviors was also reported [4]. The global efforts to combat syphilis mostly focus on screening and treatment of pregnant women. However, this strategy has difficulty to be implemented in resource-constrained countries where access to health services is limited. Lack of intervention measures for syphilis in turn makes those areas be a breeding ground and leaves the infection to gain significant public and clinical impacts [5].

Mainly as a result of shared transmission routes and due to their adverse interaction, syphilis – human immunodeficiency virus (HIV) co-infection has been a public health problem. More specifically, syphilis causes genital ulcer and facilitates HIV entry and shading. Besides, it induces immune activation and favor viral replication, which in turn accelerate HIV transmissibility [6,7]. In a systematic review of literatures from several regions of the world, a median point-prevalence of syphilis among HIV–infected patients was shown to be 9.5% [8].

In Ethiopia, a combination of social stigma and associated underreporting, their asymptomatic nature, and lack of diagnostic facilities make the health and socio-economic impacts of sexually transmitted infections (STIs) unknown [9]. But, small scale studies conducted in different localities of the country reported syphilis prevalence that ranged from 1% to 10.9% in diverse risk groups including pregnant women, blood donors, street dwellers and elderly people [10-13]. Moreover, a syphilis prevalence of 9.8% among HIV–infected patients in Addis Ababa was eightfold higher than the rate for HIV non–infected individuals [14].

Having understood the dangerous combination of syphilis and HIV, health care settings in developed nations provide routine and regular syphilis screening and treatment services for HIV–infected patients [15,16]. By contrast, syphilis is often left undiagnosed in people with HIV infection in Ethiopia as routine testing is not a part of the national guidelines [17]. Of course, the prevalence of syphilis in the particular risk group has to be determined first in order to decide whether the infection deserves priority for intervention. Moreover, as to what risk behaviors expose HIV–infected people to syphilis need to be well defined, on the basis of which effective intervention strategies could be designed.

In Ethiopia, the epidemiology of syphilis–HIV co-infection is not well studied; nor is the consequence of their adverse interaction thoroughly elucidated. We previously reported syphilis sero-prevalence in HIV infected individuals in a hospital in Addis Ababa [14]. However, no difference in syphilis prevalence by various risk exposure status was shown, which may be due to the weaker statistical power of that study. Moreover, in Ethiopian context where a varying prevalence of HIV infection by geographical region is documented [18], as to what the epidemiology of syphilis-HIV co-infection in each region looks has not been well defined. In the absence of a large nationwide wide data, small scale studies in various localities could generate valuable information for planning interventions. The present study is, therefore, aimed at determining the prevalence and risk factors of syphilis among HIV–infected people in southern Ethiopia so that the need to provide regular syphilis screening for HIV- infected population would be discussed.

## Methods

A cross-sectional study was conducted at Hawassa Teaching and Referral Hospital from January to May, 2014. The hospital is situated in Hawassa, the capital city of the Southern Nations, Nationalities and Peoples’ Regional state in Ethiopia, and it is the largest public hospital in the region. HIV-infected patients monitor their disease status in the antiretroviral therapy (ART) clinic of the hospital. Clinical and immunological assessments (CD4+ T– cell count) at enrollment and at three-monthly follow-up visits are taken to determine eligibility of patients for ART. Those who start receiving ART are monitored on a monthly basis until they show good treatment adherence, and on three-monthly basis, thereafter. There were 7500 HIV–infected people attending the ART clinic; of which, 4100 were taking ART. These patients are not routinely screened for syphilis, and only those with clinical indications of the disease are tested.

The study population consisted of all HIV–infected individuals who got immunological and biochemical testing during the study period. Sample size was estimated to be 763 using single population proportion formula, assuming 9.8% syphilis prevalence in HIV–infected patients [14]; 2% precision and 95% level of confidence. But, in attempt to enhance the statistical power of detecting rate difference by exposure status, we investigated a total of 993 consecutive patients, prospectively. Individuals excluded from the study were children < 15 years of age, as they were fewer in number; those who took syphilis treatment, as reactive non–treponemal test result might not remain after treatment; and those with a CD4+ T–cell count ≤ 50 cells/μl because of the unreliability of serological tests in a state of severe immunosuppression.

Counselor nurses interviewed the study participants using structured questionnaires on socio-demographic and other risk factors such as history of blood transfusion, age at sexual debut, multiple sexual partner (more than one sexual partner), traditional procedures (scarring, tattooing, bloodletting), circumcision, current condom use, excessive alcohol or drug use during sexual activity, and history of STIs. Blood samples were collected and screened for syphilis using the non-treponemal serologic test, rapid plasma reagin (RPR) test (Linear Chemicals SL, Barcelona Spain). Sera tested positive by RPR tests were confirmed using treponemal test, modified *Treponema pallidum* haemagglutination assay (TPHA) (Syphicheck–WB, Qualpro Diagnostics, India). Laboratory testing was carried out according to the directions of the manufacturers and all tests were run against the positive and negative controls. Only those samples positive by both RPR and TPHA were considered to have syphilis [19].

Data was analyzed using SPSS Version–16, and results were summarized using descriptive statistics. Pearson’s Chi-square test and Student’s *t*–test was used to evaluate differences between proportions and means, respectively. Multivariable logistic regression analysis was performed taking those socio-demographic and risk behavior factors found to be significantly associated with syphilis in bivariate logistic regression analysis. Odds ratio (OR) was used as a measure of the strength of association, and a p-value < 0.05 was considered to be statistically significant. The study was approved by the Ethics Committee of the College of Medicine and Health Sciences, Hawassa University. Written informed consent was obtained from all adult study participants. Assent was obtained from children under the age of 18 years in addition to a written consent from their parents or guardians. Any information obtained during the study was kept with utmost confidentiality. Study participants were tested for syphilis free of charge; and those found to be infected were managed by doctors.

## Results

Out of 1013 HIV–infected patients approached during the study period, 21 (2.1%) were excluded because 2 patients took syphilis treatment, 1 refused to participate, 3 were children aged < 15 years, and 15 had CD4+ T–cell count ≤ 50 cells/μl. Thus, data from 993 patients was considered for analysis. Majority of the study participants (83.9%) were ART–users, and had received the treatment for median duration of 48 months (range, 1–120 months). Their median CD4+ T–cell count was 429 cells/μl (range, 51–1614 cells/μl), and 36.9% of the participants had counts ≥ 500 cells/μl. Men accounted for 39.8% of the participants, with male to female ratio 0.66:1. Their mean age was 33.7 years (standard deviation (SD), 9; range, 15 – 75 years), and substantial number (42%) were in the age category 30 – 39 years. A respective 20.6% and 41.8% of the participants were merchants and had only secondary school level education. Higher proportion of the participants was currently married; but those who ever divorced or widowed were 20.4% (Table 1).

**Table 1:** **Syphilis infection in relation to socio-demography in HIV infected individuals in southern Ethiopia, 2014**

**Characteristics**	**Number (%) tested**	**Number (%) positive**	**Crude odds ratio (95% CI)**	**Adjusted odds ratio (95% CI)**	**p-value**
Residence					
Rural	59 (5.9)	1 (1.7)	1		
Urban	934 (94.5)	71 (7.6)	4.7 (0.65-34.9)	-	-
Sex					
Female	598 (60.2)	34 (5.7)	1	1	
Male	395 (39.8)	38 (9.6)	1.8 (1.1-2.86)	2.2 (1.22-3.87)	0.007
Age (years)					
<20	13 (1)	0	-	-	-
20-29	330 (33.2)	16 (4.8)	1	1	
30-39	417 (42)	31 (7.4)	1.6 (0.85-2.93)	1.7 (0.9-3.3)	0.100
40-49	161 (16.2)	9 (6.2)	1.3 (0.58-2.93)	1.3 (0.53-3.0)	0.598
≥50	72 (7.3)	15 (20.8)	5.2 (2.4-11)	3.9 (1.7-8.98)	0.001
Occupation					
Employee	194 (19.5)	10 (5.2)	1		
Daily laborer	114 (11.5)	14 (12.3)	2.5 (1.1-6)		
Student	31 (3.1)	2 (6.5)	1.2 (0.26-6.1)		
Merchant	205 (20.6)	11 (5.4)	1 (0.43-2.52)	-	-
Farmer	33 (3.3)	2 (6.1)	1.2 (0.25-5.68)		
House maid	47 (4.7)	3 (6.4)	1.3 (0.33-4.75)		
Jobless	144 (14.5)	14 (9.7)	1.9 (0.85-4.6)		
Self-employed	147 (14.8)	9 (6.1)	1.2 (0.48-3)		
others	78 (7.9)	7 (9)	1.8 (0.67-4.95)		
Educational status					
No-formal	136 (13.7)	11 (8.1)	3.2 (1.01-10.4)	4.7 (1.37-16.1)	0.014
Primary school	291 (29.3)	39 (13.4)	5.7 (1.99-16.2)	7.8 (2.63-23.2)	0.000
Secondary school	415 (41.8)	18 (4.3)	1.7 (0.56-5)	2.0 (0.65-6.24)	0.223
Certificate and above	151 (15.2)	4 (2.6)	1	1	
Marital status					
Married (never D/W)	468 (47.1)	32 (6.8)	2 (0.61-6.86)		
Married (previous D/W)	202 (20.4)	16 (7.9)	2.4 (0.68-8.89)		
Never married	139 (14)	10 (7.2)	2.2 (0.58-8.12)	-	-
Divorced (D)	97 (9.8)	11 (11.3)	3.6 (0.97-13.3)		
Widowed (W)	87 (8.8)	3 (3.4)	1		
CD4+ T-cell count/μL					
<200	125 (12.6)	8 (6.4)	1.1 (0.47-2.47)	-	-
200-349	230 (23.2)	17 (7.4)	1.3 (0.65-2.4)		
350-499	271 (27.3)	25 (9.2)	1.6 (0.88-2.89)		
≥500	366 (36.9)	22 (6)	1		
ART					
Yes	833 (83.9)	54 (6.5)	1	1	
No	160 (16.1)	18 (11.2)	1.8 (1.04-3.2)	2.2 (1.22-4.1)	0.009

ART, antiretroviral therapy.

Sera from 117 participants (11.8%) were found reactive by RPR test; 61.5% of which were tested positive by TPHA. Thus, the overall sero-prevalence of syphilis in HIV–infected participants was calculated to be 7.3% (95% CI 5.7%- 9%). As presented in Table 2, the rate of seropositive syphilis was significantly higher among ART–naïve HIV–infected participants (11.2%; 95% CI 6.3%-16.1%) compared with those ART–users (6.5%; 95% CI 4.8%-8.1%) (p = 0.03). RPR reactivity was not found to be influenced by ART status of the participants (11.2% versus 14.4%; p = 0.267). RPR–reactive sera more likely tested TPHA positive in ART–naïve participants (78.3%) than ART–users (57.4%) though the difference was marginally non–significant (p = 0.06).

**Table 2:** **Syphilis serological tests in HIV-infected individuals in southern Ethiopia, 2014**

**Syphilis test**	**Total tested**	**Number (%) of positive**	**HIV-infected participants**	
			**ART users**	**ART naïve**
			**Tested + Ve (%)**	**Tested + Ve (%)**
RPR	993	117 (11.8)	833 94 (11.3)	160 23 (14.4)
TPHA	117	72 (61.5)	94 54 (57.4)	23 18 (78.3)
Syphilis sero-positivity	993	72 (7.3)	833 54 (6.5)	160 18 (11.2)

HIV, Human immunodeficiency virus; RPR, rapid plasma regain; TPHA, Treponema pallidum haemagglutination assay; ART, antiretroviral therapy; +Ve, positive; −Ve, negative.

The exposure of HIV–infected participants to different risk factors of syphilis is summarized in Table 3. A statistically significant difference in mean age at sexual debut was observed by gender, which was 22.8 years (SD, 4.3) for men and 19.1 years (SD, 4.2) for women (p < 0.001). Study participants who reported had 2 to 5 number of life time sex partners accounted 48.2%. Most respondents had never used excessive alcohol (83.6%) or any drug (90.5%) during sexual activity. Only 28.3% of the respondents reported they always used condom currently, and 23.9% ever had been treated for STIs. Histories of tattooing (7.9%), unsafe injection (7.6%) and scaring (7.4%) were also reported by the participants.

**Table 3:** **Syphilis infection in relation to its risk factors in HIV infected individuals in southern Ethiopia, 2014**

**Characteristics**	**Number (%) tested**	**Number (%) positive**	**Crude odds ratio (95% CI)**	**Adjusted odds ratio (95% CI)**	**p-value**
History of blood transfusion					
No	957 (96.4)	65 (6.8)	1	1	
Yes	36 (3.6)	7 (19.4)	3.3 (1.39-7.9)	3.9 (1.5-10.4)	0.005
Unsafe injection					
No	918 (92.4)	65 (7.1)	1		
Yes	75 (7.6)	7 (9.3)	1.4 (0.59-3.06)	-	-
Circumcised					
No	27 (2.7)	1 (3.7)	1		
Yes	966 (97.3)	71 (7.3)	2.1 (0.28-15.4)	-	-
Traditional procedures					
No	825 (83.1)	57 (6.9)	1.9 (0.57-6.1)	1.7 (0.49-5.7)	0.404
Scaring	73 (7.4)	11 (15.1)	4.4 (1.19-16.6)	3.3 (0.84-12.9)	0.088
Blood letting	17 (1.7)	1 (5.9)	1.6 (0.15-16)	1.3 (0.12-15.1)	0.814
Tattooing	78 (7.9)	3 (3.8)	1	1	
Age at sexual debut (years)					
<20	479 (46.2)	36 (7.8)	0.84 (0.51-1.4)	-	-
20-29	495 (49.8)	33 (6.7)	0.98 (0.28-3.3)		
≥30	39 (3.9)	3 (7.7)	1		
Sex partners					
1	479 (48.2)	31 (6.5)	2.3 (0.3-17.3)	-	-
2-5	480 (48.3)	40 (8.3)	3 (0.4-22.5)		
>5	34 (3.4)	1 (2.9)			
Alcohol during sex					
No	830 (83.6)	59 (7.1)	1.1 (0.53-2.2)	1.5 (0.68-3.2)	0.321
Sometimes	151 (15.4)	10 (6.6)	1	1	
Often	12 (1.2)	3 (25)	4.7 (1.1-20.1)	3.5 (0.75-16.3)	0.110
Drug during sex					
No	899 (90.5)	65 (7.2)	1		
Sometimes	86 (8.7)	6 (7)	0.96 (0.4-2.3)	-	-
Often	8 (0.8)	1 (12.5)	1.8 (0.22-15)		
Condom use					
No	486 (48.9)	34 (7)	1		
Sometimes	226 (22.7)	16 (7.1)	1 (0.55-1.88)	-	-
Always	281 (28.3)	22 (7.8)	1.1 (0.64-3.96)		
STIs					
No	756 (76.1)	58 (7.7)	1.3 (0.72-2.42)	-	-
Yes	237 (23.9)	14 (5.9)	1		

STIs, sexually transmitted infections.

In bivariate analysis, the distribution of syphilis was shown to be influenced by ART status, sex, age, and level of education (Table 1). Similarly, history of blood transfusion, traditional procedures, and excessive use of alcohol during sexual activity were associated with syphilis (Table 3). There was no significant difference in median CD4+ T–cell count by syphilis sero–status in either ART groups nor did the cell count influence the rate of syphilis. Other factors such as residence, occupation, marital status, unsafe injection, circumcision, age at sexual debut, having multiple sexual partners, use of drug during sexual activity, current condom use practice were not associated with syphilis.

In further analysis, after adjustment for those significantly associated variables, using multivariate logistic regression, the association of syphilis with ART status remain significant where ART non–users were more infected compared with ART–users (OR = 2.2; 95% CI 1.22 – 4.1; p = 0.009). Men were also found to have twofold higher risk of seropositive syphilis than women (OR = 2.2; 95% CI 1.22 – 3.87; p = 0.007). The rate of seropositive syphilis was shown to increase with age, with the highest risk at age ≥ 50 years compared with those < 30 years (OR = 3.9; 95% CI 1.7 – 8.98; p = 0.001). The education level of participants also influenced the rate of infection where those with no formal education (OR = 4.7; 95% CI 1.37 – 16.1; p = 0.014) or with primary school level education (OR = 7.8; 95% CI 2.63 –23.2; p < 0.001) had higher odds of infection compared with those having at least a certificate. Moreover, a history of blood transfusion raised the odds of having syphilis compared with those with no history of transfusion (OR = 3.9; 95% CI 1.5 – 10.4; p = 0.005). However, the association of syphilis with traditional practice or using excessive alcohol during sexual activity did not remain significant in multivariate analysis.

## Discussion

The failure to provide screening and treatment of syphilis for HIV–infected population leaves the infection to spread further and become clinically consequential as well as to fuel HIV transmission. As part of effort to describe syphilis epidemiology in Ethiopia and bring the problem to the attention of public health officials and decision makers for possible interventions, we estimated syphilis prevalence and associated risk factors in HIV–infected people. The study showed that the sero-prevalence of syphilis was 7.3%. The rate of infection was significantly higher among participants who were ART naive, men, older than 50 years of age, had only primary school level education. Moreover, the infection was shown to predominate in those with a history of blood transfusion. But, syphilis was not found to be associated with variables such as level of CD4+ T-cell count, having multiple sexual partners, and current condom use practice.

The sero-prevalence in the current study was in agreement with our previous result in HIV- infected patients in Addis Ababa where an infection rate of 9.8% was reported [14]. A contrasting lower [20,21] and higher [22,23] rates of seropositive syphilis were shown in HIV–infected heterosexuals in various African cities. In developed nations where regular screening for syphilis is provided for HIV infected individuals, prevalence lower than 1% was shown [16]. Similar practices in developing countries may reduce the clinical and public health significance of syphilis.

Although the predominance of syphilis among ART–naive patients was in agreement with a report from Cameroon [23], it contrasted our previous finding that showed no appreciable difference [14]. But, ART–naïve patients might recently register to the ART clinic where a comprehensive HIV care is given; thus, they were yet with untreated syphilis and/or with risky sexual behaviors. Previous reports indicated that sero-prevalence of syphilis increased with age [11,13,14,22,23] and complement the current higher rate of infection among participants older than 50 years of age. This is perhaps due to the risk of exposure to syphilis increased with time or there was higher exposure during early days with the emergence of HIV epidemic. The preponderance of syphilis among men compared to women in our study was concordant with findings reported in heterosexual population elsewhere [20,23]. In contrast, similar rate of infection by gender was also shown in Ethiopia [13,14] though those studies had no strong statistical power to be able to detect rate difference. In Ethiopian context where HIV is contracted primarily by heterosexual exposure and women are disproportionality infected, as to what risk behaviors predisposed men to contract syphilis require further investigation. It was noticed that the sero-prevalence of syphilis decreased with increasing level of education as also shown previously [14]. The higher odds of infection in those participants who reported had a history of blood transfusion was in agreement with a result shown elsewhere [24]. This result may point out the need to ensure our blood bank centers take adequate safety measures that reduce transfusion-transmitted syphilis.

However, the lack of association between a history of multiple sexual partners and syphilis in our previous [14] or current study contrasted a result by Moges *et al*. that showed a six-fold rate difference among street dwellers. But, regardless of having multiple sexual partners, our study participants had already been exposed to HIV infection during which syphilis could simultaneously be contracted. The decreased median CD4+ T-cell count among people with syphilis–HIV co-infection compared with HIV mono-infected individuals shown by others [25,26] was not observed in the current study. Nor sero-prevalence of syphilis was significantly influenced by the level of CD4+ T-cell count.

This study has some limitations in light of which results need be interpreted. First, as a hospital based study that used a non-probability sampling method, selection bias may be introduced that hinder the generalizability of the result to all HIV–infected population in the study area. But, the relatively larger sample size that we used may reduce the effect of bias and ensure quality of the generated data. Second, the potential for false negative results owing to prozone reactions and reduced sensitivity of the non-treponemal tests in primary as well as late latent syphilis may lead to underestimation of rate of infection. Moreover, positive results with non-treponemal and treponemal tests may not necessarily indicate disease activity as there is possibilities for false-positive reaction.

## Conclusion

This study showed high sero-prevalence of syphilis among HIV–infected individuals, especially among those participants who were ART-naive, men, older than 50 years of age, had only primary school level education, and ever had received blood transfusion. The observed high rate of infection warrants the need to target HIV–infected population with syphilis interventions in order to reduce its clinical and public health impacts. As part of this effort, integrating syphilis screening and treatment service with HIV/AIDS care is critically needed and could limit the clinical consequences of untreated syphilis as well as its adverse impact on HIV transmission.

## Abbreviations

HIV: Human immunodeficiency virus
RPR: Rapid plasma regain
TPHA: Treponema pallidum haemagglutination assay
ART: Antiretroviral therapy
STI: Sexually transmitted infection
OR: Odds ratio
SD: Standard deviation
